# Computational model of human ventilation for electrical stimulation following cervical spinal cord injury

**DOI:** 10.1186/1471-2202-15-S1-P133

**Published:** 2014-07-21

**Authors:** Brian K Hillen, Ranu Jung

**Affiliations:** 1 Department of Biomedical Engineering, Florida International University, Miami, Florida, 33174, USA

## 

**Figure 1:**
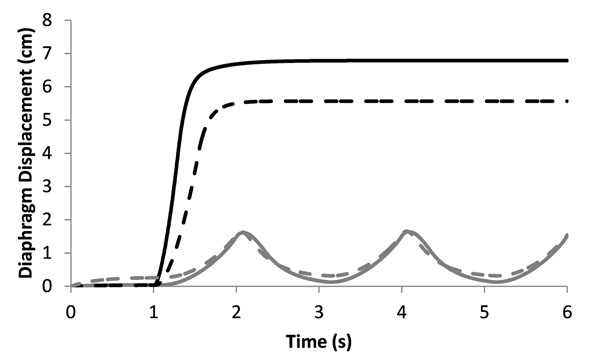
Diaphragm displacement during tidal breathing (grey lines) and maximal inspiration (black lines). Uninjured curves (solid lines) reproduce published experimental data. When simulating electrical stimulation (reverse recruitment) and fatigue (a 50% reduction in maximal muscle force), tidal breathing results are similar to uninjured simulations due to increased force generated by the larger motor units being recruited first (dashed lines).

Towards the development of an implantable stimulator for respiratory pacing, we have developed a computational model of the human ventilatory system to simulate breathing maneuvers. Following cervical spinal cord injury, control of breathing is lost or diminished. If the loss is severe, patients are dependent on mechanical ventilators or electrical stimulators to regain functional breathing. Existing electrical stimulation based respiratory pacing systems typically activate only the diaphragm and cannot adapt to muscle fatigue, changes in respiratory demands, or changes in electrode quality over time. This model will be used to test stimulation controllers that can adapt to such conditions. The model was developed in Simulink/SimMechanics implementing the physiologically realistic muscle model from MSMS [1,2] using published parameters for muscle geometry [3,4]. Diaphragm geometry was modeled as a pulley system in one dimension. Ventilatory compliance was modeled as a damped spring with non-linear stiffness. Integrated phrenic drive was modeled as either a spaced sawtooth (tidal breathing) or a step function (maximal inspiration). The model was able to reproduce tidal breathing and maximal inspiration for uninjured subjects (see Figure 1). When simulating electrical stimulation without fatigue, maximal inspiration was similar but tidal breathing was higher due to the recruitment of larger motor units first. When simulating electrical stimulation as well as fatigue (50% reduction in maximal force and a decrease in the power of the fast motor units), tidal breathing was similar to the uninjured case (see Figure 1). Thus, even with fatigue, electrically stimulated muscles are strong enough to produce the submaximal contractions needed for tidal breathing. In the future, this model will be enhanced to incorporate additional muscles of respiration as well as atrophy associated with spinal cord injury and will be used to iteratively develop adaptive controllers to achieve functional ventilation and to promote weaning from a mechanical ventilator.
